# Using the 5C Vaccine Hesitancy Framework to Elucidate and Measure Contraceptive Acceptability in sub-Saharan Africa

**DOI:** 10.9745/GHSP-D-24-00210

**Published:** 2024-12-20

**Authors:** Lotus McDougal, Caroline Deignan, Peter Kisaakye, Courtney McLarnon, Rebecka Lundgren, Shannon Pryor, Madeleine Short Fabic

**Affiliations:** a Center on Gender Equity and Health, University of California San Diego, La Jolla, CA, USA.; b Matchboxology, Cape Town, South Africa.; c Makerere University, Kampala, Uganda.; d Save the Children, Washington, DC, USA.; e U.S. Agency for International Development, Washington, DC, USA.

## Abstract

We draw lessons from immunization research by assessing the applicability of the 5C framework of vaccine hesitancy to contraceptive acceptability in sub-Saharan Africa.

## Key Messages

- There is a gap between goals of supporting reproductive agency and empowerment within family planning programs and measures able to track progress toward those goals. Commonly used family planning measures are beset by imprecise terminology and oversimplification and are often not person centered.
- Measuring contraceptive acceptability, which offers a means of understanding if and how contraceptive desire translates into contraceptive demand, requires a conceptual framework identifying the diverse factors that contribute to it.
- In assessing how the 5C framework of vaccine hesitancy (confidence, calculation, constraints, complacency, and collective responsibility) may inform our conceptualization of contraceptive acceptability in sub-Saharan Africa, we found that it has relevance, with confidence, calculation, and constraints appearing particularly salient.
- However, contraceptive acceptability is informed by additional social and gendered factors, including gender roles, gendered power dynamics, and social norms.
- This work represents a first step toward developing and testing a measure of contraceptive acceptability that can more precisely diagnose and support the achievement of self-determined reproductive goals and track progress toward those goals within family planning programs and at individual, community, and population levels.

See related article by Short Fabic and Tsui.

## INTRODUCTION

Thirty years after the International Conference on Population and Development (ICPD), ICPD-related themes of reproductive rights, individual agency, and reproductive empowerment remain central to family planning (FP) efforts.^1^^–^^3^ However, measurement of these themes remains a challenge, in large part because of their complexity and contextual nuance.^4^^–^^7^ As the FP community continues to move toward ensuring alignment of goals, indicators, and measurement approaches with these ICPD themes, we see opportunities to overcome some of our long-standing measurement challenges by learning from other health sectors.

Here, we present results from our recent literature review, which builds from a suggestion made by the World Health Organization’s Strategic Advisory Group of Experts on Immunization to consider that “reproductive health decisions are a behavioral phenomenon like vaccine decisions.”^8^ Our interest is in applying the lessons learned from vaccination hesitancy research to inform our understanding and measurement of the drivers of contraceptive acceptability, defined as the degree to which an individual perceives contraception as (un)acceptable for pregnancy prevention. We examine these relationships at individual and community levels, including how such drivers influence contraceptive intentions, decisions, use, and non-use.

We hypothesize that such a measurement lens and approach could deepen our collective understanding of reproductive agency and better inform person-centered programming, which prioritizes people’s individual needs, preferences, and values.^9^^,^^10^ These conceptual foci underscore a fundamental difference between the fields of immunization and FP, namely, that there is not a desired choice or behavior within a person-centered, agency-focused approach to FP beyond supporting and enabling an individual to achieve the unique reproductive goals that they have chosen.

We hypothesize that using a vaccine hesitancy measurement lens and approach could deepen our collective understanding of reproductive agency and better inform person-centered programming.

The application of a vaccination-derived paradigm of hesitancy and acceptability to individual perceptions of contraceptive use is a novel framing. To begin to address this evidence gap, an article in *GHSP* presents the motivation and grounds for a contraceptive-focused adaptation of the 5C framework.^11^ In that article, which complements this work, Short Fabic and Tsui explore how the 5C framework of vaccine hesitancy might translate to contraceptive acceptability based on existing research from sub-Saharan Africa. In tandem, these 2 articles represent the initial steps we have taken in our attempt to understand the applicability of a vaccine hesitancy framework and related research to the FP sector. We use these results to examine whether contraceptive acceptability merits further pursuit as a person-centered measure (i.e., a measure that prioritizes people’s individual needs, preferences, and values rather than externally defined goals and targets) within broader FP measurement efforts.

## MEASURING THE COMPLEXITY OF CONTRACEPTIVE ACCEPTABILITY

Programs and policies focused on reproductive agency and empowerment need measures built around the same goals and paradigms to track progress, identify successes, and diagnose challenges. The existing landscape of agency and empowerment-focused FP measurement is limited in many ways, including a lack of widespread conceptual agreement, constrained data collection avenues (i.e., minimal survey “real estate”), and a lack of clarity from governments and other stakeholders on what action(s) to take in response to such information.^3^^,^^12^^–^^14^ For example, the indicator “informed sexual and reproductive decision-making” has the broad consensus needed to merit inclusion in the Sustainable Development Goals. However, the data used to track progress (generally collected through the Demographic and Health Surveys) lack key information related to agency and empowerment, including the depth and breadth of diverse respondent’s sexual and reproductive knowledge and preferences, respondent’s decision satisfaction, and broader supply-side factors informing the contexts in which decision-making occurs.^7^^,^^15^^,^^16^ Moreover, because interventions to affect women’s increased decision-making engagement are not straightforward, unlike, for example, interventions to increase contraceptive availability, meaningful programmatic response to indicators focused on decision-making is often limited.^17^^–^^19^ Other widely used measures, such as unmet need for FP and demand satisfied for FP, do not directly assess an individual’s stated need or demand for contraception, and as a result, they are widely criticized as being researcher derived rather than person centered.^4^^–^^6^ These measures are operationalized as a dichotomy—a woman’s “need” is met or unmet, or her “demand” is present or absent.^7^^,^^20^ Add to these issues terminological imprecision,^4^^,^^5^ and we end up with a set of indicators that oversimplify and potentially distort our understanding of how an individual’s contraceptive behaviors—to initiate, continue, switch, or discontinue use—align with their own preferences and reproductive goals and how such behaviors are informed and shaped by relational and contextual factors.

Understanding the multidimensional factors that influence contraceptive uptake—and lack thereof—is thus a critical piece of the complex landscape of reproductive empowerment. There have been several new measures developed in recent years, including contraceptive autonomy,^3^ reproductive agency,^21^^,^^22^ fertility norms,^23^ supply- vs. demand-side unmet need,^16^ and preference-aligned fertility management.^24^ This work showcases important advances but does not capture the full spectrum of psychological and contextual factors that influence how acceptable contraceptive use is to a given individual or community. An important lens through which to examine this willingness could be that of contraceptive acceptability. We posit that contraceptive acceptability, in tandem with reproductive agency, is one of the key factors that explains whether and how the desire to use a contraceptive method translates into demand for that method. Our conceptualizations use Short Fabic’s definitions of desire as “a wish” and demand as “desire plus ability and willingness to enact that desire,” where the desire in question is use of a given contraceptive method—by initiation, switching, or sustaining use, based on individual circumstances.^5^ Defining and measuring contraceptive acceptability has the potential to offer substantial and actionable insights into programmatic gaps and opportunities to better enable individuals to achieve their reproductive goals.

## METHODS

### Vaccine Hesitancy

Development of a theoretical framework and measure of contraceptive acceptability may be informed by work done by immunization social and behavior change practitioners and researchers, who have several decades of experience conceptualizing and measuring vaccine hesitancy.^11^^,^^25^^–^^27^ In this article, we opted to use the term “contraceptive acceptability” rather than “contraceptive hesitancy” to better reflect a position of nonjudgement within a person-centered framing.

As the World Health Organization has recognized, both vaccine and contraceptive “hesitancy” manifest in an intersectional network of individual, interpersonal, social, and structural influences.^8^^,^^28^ Building from existing conceptual models and theoretical underpinnings of vaccine hesitancy,^28^^–^^30^ as well as well-known behavioral theories, including the Health Belief Model^31^^,^^32^ and the Theory of Planned Behavior,^33^ Betsch et al. developed the 5C framework of psychological antecedents of vaccination.^34^ This framework and the associated measure encompass confidence (in vaccines and associated delivery systems), calculation (gathering and assessing information), constraints (both structural and psychological), complacency (in perceived risk associated with vaccine-preventable disease), and collective responsibility (to protect others).^34^ We took these 5 constructs that comprise vaccine hesitancy, developed corollaries for contraceptive acceptability through author discussion and consensus, and examined published literature to examine the salience of this adaptation within existing research. Our proposed translation is summarized in the Figure, which we will further hone as we analyze the forthcoming results of formative research. This framing examines contraceptive use from a lens of pregnancy prevention; while there are other reasons that individuals may choose to use contraception,^5^ they are outside the scope of this analysis.

**FIGURE:**
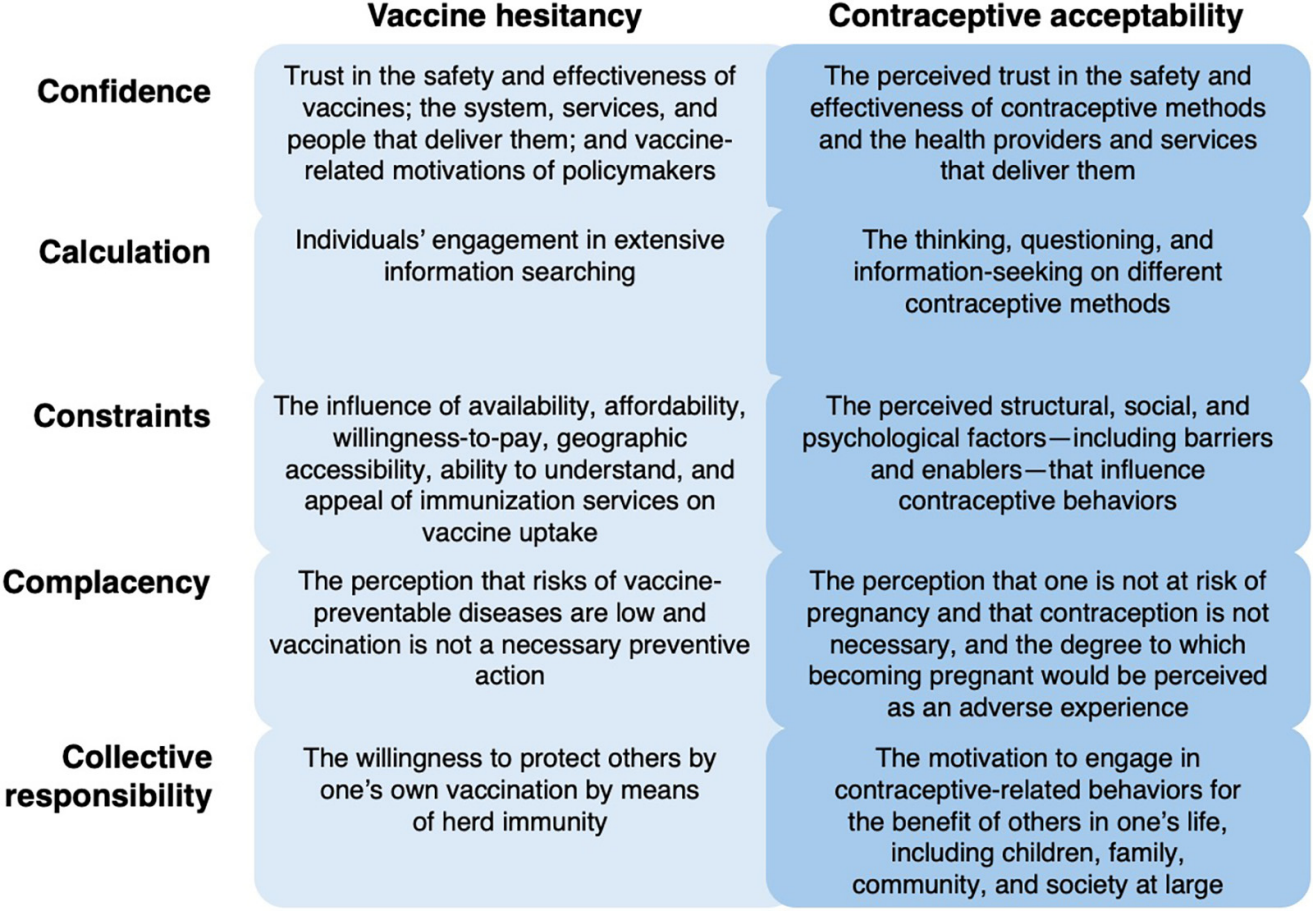
Summary of 5Cs of Vaccine Hesitancy^a^ and Proposed Adaptation to Contraceptive Acceptability ^a^ Betsch et al.^34^

### Geographic Setting

There is a clear need to elucidate factors influencing contraceptive acceptability in sub-Saharan Africa, a region which bears the highest rates of maternal mortality in the world,^35^ coupled with some of the lowest levels of women’s engagement in sexual and reproductive health decision-making^36^ and of contraceptive use.^37^ Within East, Central, West, and Southern Africa (hereafter referred to as sub-Saharan Africa), there are substantial variations in contraceptive prevalence,^37^ reflecting the importance of the individual, interpersonal, social, and structural factors that shape whether and how the desire to use a contraceptive method translates into demand for that method.^38^ This analysis is limited to sub-Saharan Africa to enable a more focused conceptual review and subsequent research that is regionally informed and relevant. Understanding contraceptive acceptability globally is an important next step in this work, particularly given the heterogeneity in contraceptive methods and determinants across regions,^37^^,^^39^ but is beyond the purview of the current research.

### Considering the Existing Evidence on the 5Cs in sub-Saharan Africa

This commentary builds from a recent literature review examining published research related to contraceptive acceptability in sub-Saharan Africa.^40^ The review searched 3 scholarly databases (PubMed, EBSCOHost, and Cochrane) for articles that explored drivers of contraceptive acceptability and uptake. Eligible articles were published in English between 2010 and 2022 and focused on 1 or more sub-Saharan African countries. Articles that search terms returned were title/abstract screened for relevance, and eligible articles were full text reviewed by select commentary coauthors. Search terms and parameters are provided in the Supplement; full methodology is available separately.^40^ In total, search terms returned 878 articles, 262 of which were eligible for full-text review.

## RESULTS

The following summarizes existing research on contraceptive acceptability in sub-Saharan Africa across the 5Cs of vaccine hesitancy.

### Confidence

Contraceptive knowledge and trust both emerged as important factors informing contraceptive acceptability in sub-Saharan Africa. Limited fertility knowledge and awareness and misinformation about contraceptive methods and both real and perceived side effects contributed to compromised trust and uncertainty across populations and geographies.^41^^–^^44^ These factors were often described in relation to fear and were among the most identified reasons given for contraceptive uncertainty,^42^^,^^45^^–^^49^ particularly related to modern methods.^50^^,^^51^ Compromised trust was especially related to worries about delayed return to fertility and infertility; distrust in the safety and underlying purpose of contraception (e.g., that contraception will cause infertility, and this is the true intent of contraceptive services) was commonly cited.^42^^,^^43^^,^^45^^,^^50^^,^^52^^–^^55^

### Calculation

Thinking, questioning, and information-seeking often culminated in assessments of risks and benefits of contraceptive use. In sub-Saharan Africa, these assessments focused heavily on side effects, including weight gain, reduction in sexual pleasure and desire, and cancer.^42^^,^^43^^,^^45^^,^^50^^,^^52^^,^^56^^–^^58^ A related side effect that emerged prominently in the literature was that of menstrual changes, comprising heavy and persistent bleeding, as well as amenorrhea.^41^^,^^57^^,^^59^^–^^62^ These changes in menstrual patterns were often perceived as indicators of poor health and compromised fertility and were also noted as a potentially more public, if inadvertent, indication of contraceptive use.^41^^,^^57^^,^^60^^–^^62^ Concerns that contraceptive use would influence future fertility were noted in multiple settings^43^^,^^45^^,^^50^^,^^52^^,^^57^^,^^58^ and were particularly salient in contexts of normalized high fertility and stigma related to childlessness.^45^^,^^53^^,^^57^

### Constraints

Multiple structural (e.g., access, availability, and affordability), social (e.g., social pressure and stigma), and psychological (e.g., lack of desire) factors drove perceived acceptability of contraception in sub-Saharan Africa. Research highlighted limited access, affordability, and restrictive laws and policies overall, and for single and young people, provider bias and discrimination based on a client’s age or parity and fear of stigma, embarrassment, and social discomfort within FP-focused client-provider interactions.^46^^,^^48^^,^^49^^,^^53^ Additionally, challenges, such as inadequate transportation to facilities, shortage of trained providers, limited contraceptive method options, high costs, and supply shortages, further influenced contraceptive acceptability, as did religious beliefs.^42^^,^^45^^,^^46^^,^^63^ Pressure to demonstrate fertility early and often was prominent among younger women, influencing decisions to delay or avoid contraception.^64^

Multiple structural, social, and psychological factors drove perceived acceptability of contraception in sub-Saharan Africa.

### Complacency

Very little research identified sentiments that women were not at risk of pregnancy, nor the degree to which becoming pregnant would be perceived as an adverse event. Where this was discussed, perceived risk was lower among younger, healthy women.^45^ Childbearing ambivalence did not emerge strongly in this review, with only 1 study discussing the dynamic nature of ambivalence.^65^

### Collective Responsibility

While there is evidence of the importance of normative community and social influences on contraceptive acceptability,^54^^,^^57^^,^^66^^,^^67^ our literature review did not identify any research from sub-Saharan Africa describing altruistic motivations for contraceptive behaviors. This may be at least in part because, unlike with immunization, there is no “correct” choice for contraception other than a choice that is aligned with a self-determined reproductive goal. This lack of a universal, utilization-focused target may thus compromise the ability to work toward a broadly understood, altruistic goal in a given context.

### Beyond the 5Cs

While the 5Cs—particularly confidence, calculation, and constraints—were reflected in published literature exploring factors influencing contraceptive acceptability in sub-Saharan Africa, interpersonal and social factors emerged as central, cross-cutting aspects that were not encompassed within the 5C framework as originally conceptualized for vaccine hesitancy. Gender roles and gendered power dynamics were prominent in influencing contraceptive acceptability, informing pregnancy-related goals, and the agentive avenues available with which to achieve those goals. Patriarchal hegemony informed contraceptive acceptability, decision-making, and use through pervasive power imbalances;^68^^–^^70^ subverting those imbalances through male inclusion and gender transformative programming was highlighted as an important means of increasing women’s ability to achieve their reproductive goals.^69^^–^^71^ Social norms manifested in multiple areas that influenced contraceptive acceptability, including demonstration of fertility, support for larger family sizes,^57^^,^^67^^,^^72^ and fear of sanctions for using contraception.^42^^,^^73^ These norms were heavily influenced by social networks for both women and men.^54^^,^^57^^,^^66^

## DISCUSSION

### Implications for Family Planning Programming and Measurement

Findings from the literature review affirm our hypothesis that the 5C framework of vaccine hesitancy has applicability to the FP field, particularly as we embark on work to measure, understand, and develop programming responsive to contraceptive acceptability. Confidence, calculation, and constraints emerged clearly in published literature on factors influencing contraceptive decisions in sub-Saharan Africa, while complacency and collective responsibility were less prominent. Additionally, findings indicate that a contraceptive acceptability framework must go beyond the 5Cs to include gender roles and gendered power dynamics. While these social and normative factors are important components of vaccine hesitancy,^74^^–^^76^ they are not included in the 5C framework, which explicitly focuses on individual psychological concepts.^34^ This review of contraceptive acceptability—and hesitancy—in sub-Saharan Africa underscores the centrality of interpersonal relationships and social structures in influencing individuals’ perceptions of contraceptives,^77^^,^^78^ suggesting that a framework that omits consideration of these complex influences may be inadequate. Clearly, additional work is needed beyond the adaptation proposed in the Figure. We are embarking on that by conducting formative research and measure development—including cognitive testing—to hone a framework and measure of contraceptive acceptability, which we plan to test in longitudinal studies beginning later this year.

Findings indicate that a contraceptive acceptability framework must go beyond the 5Cs to include gender roles and gendered power dynamics.

In the meantime, we recognize that the 5C vaccine hesitancy framework has informed various measurement efforts to capture person-level determinants of vaccine behaviors and practices.^34^^,^^74^^,^^79^ This provides a roadmap to consider both individual factors (e.g., knowledge, beliefs, attitudes and motivation about health, trust in health system, experience with vaccination, and peer influence) and perceptions of vaccine-specific and vaccination-specific structural determinants (e.g., cost, vaccination schedule, mode of administration). The 5C—and other vaccine hesitancy frameworks—have not only been used to inform measurement but to translate those insights into vaccine programming, most recently for the COVID-19 vaccine efforts.^80^^,^^81^ Applying the 5C and other vaccine frameworks has allowed the immunization field to identify which factors influence, mediate, or have little bearing on vaccine intentions and behaviors.^79^

If a similar measurement framework were to be successfully developed and applied to the FP sector, we would expect to have a programmatically relevant, person-level indicator for diverse users and non-users alike and for people of all genders on the determinants of contraceptive behaviors across the life course. Such information could, for example, help us to better design programs to address misalignment between fertility intentions and contraceptive behaviors; identify and mitigate the drivers of method discontinuation while still at risk of unwanted pregnancy; identify demand- and supply-side barriers to contraceptive uptake, continuation, and discontinuation; and understand the broader context in which an individual takes action to achieve their reproductive goals. This broader context is an essential component in considering contraceptive demand (and use or non-use) as service-related factors (such as access, cost, and quality of care); gendered power dynamics (such as restrictions on freedom of movement or reproductive coercion); and cultural factors (such as religious prohibitions) all influence the ability to enact a given desire.^5^^,^^82^^–^^86^ Contraceptive acceptability offers insights into many of these aspects from an individual perspective. Though contraceptive acceptability is currently under-considered and unmeasured, results from our literature review indicate that it has substantial diagnostic potential to understand contraceptive behaviors within the full landscape in which an individual manifests their contraceptive-related desires and demands.

There are important differences between vaccination and contraceptive use,^11^ not least of which is that while, in general, universal vaccination is an agreed-upon goal, contraceptive use depends on individual intentions and desires and is deeply driven by social context. Thus, contraceptive acceptability conceptualizations must be rooted in understanding that the desired goal is not contraceptive use but rather reproductive agency. Such a contraceptive acceptability framework has the potential to support the FP community’s efforts to help individuals achieve a diversity of reproductive health goals. Applying contraceptive acceptability to FP programs and policy is a way to meet people where they are by capturing the nuances behind their contraceptive decisions across defining life course transitions. Here, it’s worth reiterating that a paramount difference between vaccine hesitancy and our conceptualization of contraceptive acceptability is that there is no “correct” behavior; in other words, the focus is not “Why aren’t you…?” Rather, the focus is on understanding the drivers of myriad behaviors with the goal of ensuring that individuals have the freedom to decide if, when, and with whom to have children and the knowledge and means necessary to achieve their unique goals. Importantly, this focus on reproductive agency is distinct from policies and goals focused on influencing fertility levels (including more than 70% of countries in sub-Saharan Africa aiming to lower fertility rates^87^) or those focused on contraceptive use, including Sustainable Development Goal indicator 3.7.^88^ The relationship between measures and goals focused on reproductive agency vs. reproductive behaviors is complex and interrelated and will require ongoing dialogue across stakeholders to evolve and advance.

A contraceptive acceptability framework can inform a standard and simple yet comprehensive measure to assess and prioritize the wide array of individual, interpersonal, social, and structural influences on contraceptive use. Currently, research and evaluation efforts must pick and choose from a multitude of measures addressing some of these factors, resulting in a piecemeal understanding and interventions and evaluation efforts that miss the mark. With a more holistic understanding, combined with community consultation, programs and policies can more effectively address the multilayered barriers that prevent individuals from achieving their reproductive goals. Importantly, operationalizing this measure as a scale rather than a dichotomous “yes” or “no” question will allow a more realistic reflection of the degree of contraceptive acceptability that an individual feels at a given point in time.^89^^–^^92^ Enumerating this scale across a representative sample of the general population (across gender, marital status, and parity) could offer insights into demographic differences, as well as illustrate how community norms and attributes influence individual contraceptive perceptions and behaviors. This sort of analysis would also help to explicate degrees of contraceptive acceptability across different contraceptive methods. As the 5C framework may be used to understand hesitancy toward different vaccinations and vaccination types,^93^^,^^94^ so too could such a framework be used to understand acceptability of specific contraceptive methods, as well as attribute-based groupings of those methods (by type, user control, administration route, and duration). These attributes are particularly meaningful for a construct in which contributing factors are likely to vary across populations, by life events, and over time.^90^^,^^95^^–^^98^

We believe that a contraceptive acceptability measure could have clear utility to monitor and assess whether and how efforts are working to increase reproductive agency. At the population level, it could assess the cumulative effectiveness of multiple program and policy interventions over time on a variety of service delivery, interpersonal, and social factors, including social and gender norms. Such information could also be disaggregated for different subpopulations or geographies to provide deeper contextual insight. A contraceptive acceptability measure could also be used to understand and respond to issues concerning contraceptive method choice, including efforts to ensure that voluntary, safe, effective, and diverse modern methods are accessible for all who want them—including fertility awareness-based methods, hormonal methods, barrier methods, and other nonhormonal methods.

## CONCLUSION

In the 30 years since ICPD, the FP field has made remarkable advancements in fulfilling its commitments to reproductive rights, agency, and empowerment. Despite these transformative shifts, a disconnect persists in our ability to precisely and clearly measure these constructs in ways that can be scaled. Contraceptive acceptability offers an important lens through which to understand what happens between an individual having a desire to use a contraceptive method and transforming that desire into demand for that contraceptive method. Accurately conceptualizing and measuring contraceptive acceptability can amplify current person-centered measurement efforts by offering a nuanced diagnostic that FP programs can use to design, monitor, and evaluate efforts to support their clients to achieve their reproductive goals.

Contraceptive acceptability offers an important lens through which to understand what happens between an individual having a desire to use a contraceptive method and transforming that desire into demand for that contraceptive method.

Our review indicates that the 5C framework of vaccine hesitancy is relevant when considering factors influencing contraceptive acceptability in sub-Saharan Africa, with confidence, calculation, and constraints particularly salient based on published literature. However, it appears that contraceptive acceptability has influences beyond this framework, with cross-cutting factors, such as gender roles and social norms, often inhibiting women’s agency to initiate, continue, discontinue, or switch contraceptive methods of their choosing. This mirrors critiques of vaccine hesitancy framing that prioritize the role and responsibility of the individual and omit consideration of historic and social factors that inform hesitancy, including social norms.^99^^,^^100^ The prominence of these interpersonal and social factors in explaining the spectrum of contraceptive acceptability highlights the importance of acceptability as an integral facet of reproductive agency in sub-Saharan Africa, as well as the need to consider factors beyond the individual when exploring contraceptive acceptability.

The ability to set self-determined goals for if, when, with whom, and how often to become pregnant and to work to achieve those goals is a foundational connection between contraceptive desire and contraceptive use (or non-use). Contraceptive acceptability informs that desire-demand-use spectrum. Understanding the degree of acceptability and the components of acceptability that are most important for a given person, couple, or community will enable FP programs to better focus their efforts on ensuring that women have the freedom to decide if and when to have children and the knowledge and means necessary to achieve their unique goals. This, in turn, will bring us closer to realizing the guiding themes of reproductive rights, individual agency, and reproductive empowerment as articulated 30 years ago, in 1994, at ICPD.

## Supplementary Material

GHSP-D-24-00210-Supplement.pdf

## Notes

**Peer Reviewed**

**First Published Online:** November 25, 2024.
