# Supplementary material for: Using the 5C Vaccine Hesitancy Framework to Elucidate and Measure Contraceptive Acceptability in sub-Saharan Africa
Source: Glob Health Sci Pract. 2024 Dec 20;12(6):e2400210. doi: 10.9745/GHSP-D-24-00210 (PMC11666076; doi:10.9745/GHSP-D-24-00210)
Supplement: GHSP-D-24-00210-Supplement.pdf [file GHSP-D-24-00210-Supplement.pdf]

Supplement to: McDougal L, Deignan C, Kisaakye P, et al. Using the 5C vaccine hesitancy framework to elucidate and measure contraceptive acceptability in sub-Saharan Africa. *Glob Health Sci Pract*. 2024;12(6):e2400210. https://doi.org/10.9745/GHSP-D-24-00210

SUPPLEMENT. Search methodology and terms for literature review

Databases searched: PubMed, EBSCOHost, and Cochrane

Language: English

Publication date: January 1, 2010 and November 30, 2022

Search terms:

Concept	Search terms	Operator
Contraceptive	contraception, contracepti*, contraceptive method*, contraceptive device*, family planning, planned parenthood, birth control*, birth prevention method, pregnancy prevention, hormonal contracept*, conception prevention, pill*, coc*, condom*, implant*, dmpa, vasectomy, emergency contraception, btl, iud, foam, jelly, ring, steriliz*	OR
AND		
Hesitancy	hesit*, refus*, disinclin*, decline, reluct*, reservation*, disinclination, unwilling*, uncertain*, mistrust, skeptic*, intent*, indecision, indecis*, caution, doubt, fence-sitting, delay, cynic, pause, anx*, barrier*, fear*, oppos*, choice*, unconfid*	OR
AND		
Sub-Saharan Africa	Sub-Saharan Africa, east* Africa, west* Africa, Benin, Burkina Faso, Cape Verde, Cote D'Ivoire, Gambia, Ghana, Guinea, Guinea-Bissau, Liberia, Mali, Mauritania, Niger, Nigeria, Senegal, Sierra Leone, Togo, Burundi, Comoros, Djibouti, Ethiopia, Eritrea, Kenya, Madagascar, Malawi, Mauritius, Mozambique, Rwanda, Seychelles, Somalia, Somaliland, Tanzania, Uganda, Zambia, Zimbabwe, eSwatini, Lesotho, Botswana, Namibia, South Africa, Angola, Cameroon, Central African Republic, Chad, Congo, Democratic Republic of the Congo, Equatorial Guinea, Gabon, Sao Tome and Principe	OR
